# Metachronous colorectal cancer risks after extended or segmental resection in *MLH1*, *MSH2*, and *MSH6* Lynch syndrome: multicentre study from the Prospective Lynch Syndrome Database

**DOI:** 10.1093/bjs/znaf061

**Published:** 2025-04-15

**Authors:** Prospective Lynch Syndrome Database, K Zalevskaja, K Ojala, A Petrov, S Haupt, L Sunde, I Bernstein, M A Jenkins, S Aretz, M Nielsen, G Capella, F Balaguer, D G Evans, J Burn, E Holinski-Feder, L Bertario, B Bonanni, A Lindblom, Z Levi, F Macrae, I Winship, J-P Plazzer, R Sijmons, L Laghi, A Della Valle, K Heinimann, T Dębniak, R Fruscio, F Lopez-Koestner, K Alvarez-Valenzuela, L H Katz, I Laish, E Vainer, C Vaccaro, D M Carraro, K Monahan, E Half, A Stakelum, D Winter, R Kennelly, N Gluck, H Sheth, N Abu-Freha, M Greenblatt, B M Rossi, M Bohorquez, G M Cavestro, L S Lino-Silva, K Horisberger, M G Tibiletti, I do Nascimento, H Thomas, N T Rossi, L A da Silva, A Zaránd, J Ruiz-Bañobre, V Heuveline, L J Lindberg, I Gögenur, J L Hopper, A K Win, R W Haile, N Lindor, S Gallinger, L Le Marchand, P A Newcomb, J Figueiredo, D D Buchanan, S N Thibodeau, M v Knebel Doeberitz, M Loeffler, N Rahner, E Schröck, V Steinke-Lange, W Schmiegel, D Vangala, C Perne, R Hüneburg, S Redler, R Büttner, J Weitz, M Pineda, N Duenas, J Brunet Vidal, L Moreira, A Sánchez, J Castillo-Iturra, E Hovig, K Green, F Lalloo, J Hill, E Crosbie, M Mints, Y Goldberg, D Tjandra, S W ten Broeke, R Kariv, G Rosner, A Jain, P Shah, M Shah, F Neffa, P Esperon, W Pavicic, G T Torrezan, T Bassaneze, C A Martin, K Pylvänäinen, G Möslein, A Lepistö, J-P Mecklin, L Renkonen-Sinisalo, J R Sampson, M Dominguez Valentin, P Møller, T T Seppälä

## Abstract

This first prospective observational study evaluates the impact of extended versus segmental colorectal surgery on the risk of metachronous colorectal cancer (CRC) in patients with Lynch syndrome, analyzing data from the Prospective Lynch Syndrome Database version 5. Extended resection significantly reduced the risk of metachronous CRC in path_MLH1, path_MSH2, and path_MSH6 carriers compared to segmental resection.

## Introduction

Lynch syndrome (LS) is an autosomal dominant cancer predisposition syndrome caused by a pathogenic germline variant of one of the DNA mismatch repair (MMR) genes (*MLH1*, *MSH2*, *MSH6*, or *PMS2*) or epigenetic silencing of *MSH2* caused by a deletion in the *EPCAM* gene^1,2^. Pathogenic MMR variant (*path_MMR*) carriers have a high lifetime risk of developing colorectal, gynaecological, urinary tract, and other cancers. LS-associated cancers have MMR deficiency, leading to microsatellite instability (MSI) in the tumours.

According to the European Hereditary Tumour Group’s latest position report, LS is now considered an umbrella term for four distinct types of LS: *MLH1* syndrome, *MSH2* syndrome, *MSH6* syndrome, and *PMS2* syndrome. These syndromes vary with regard to the age of onset of the associated cancers, sex predominance, and cancer incidence rates^3^.

Analysis of 8500 *path_MMR* carriers undergoing colonoscopic surveillance has shown that, for colon cancer, the cumulative risks at 65 years of age are 36.3%, 29.8%, 10.1%, and 2.8% in females and 48.4%, 41.5%, 12.7%, and 9.5% in males for *path_MLH1*, *path_MSH2*, *path_MSH6*, and *path_PMS2* carriers respectively. For rectal cancer, the corresponding cumulative risks at 65 years of age are 4.6%, 7.6%, 3.9%, and 2.2% in females and 6.0%, 12.6%, 5.1%, and 0% in males^4^. Colonoscopic surveillance is routinely recommended for all LS patients^5–8^. However, despite colonoscopy with the removal of adenomas every 1–3 years, colorectal cancer (CRC) incidence remains high among LS patients^9,10^.

Once colon cancer is identified, current European and American guidelines recommend considering extended colorectal surgery for *path_MLH1* and *path_MSH2* carriers, whereas segmental resection is recommended for *path_MSH6* and *path_PMS2* carriers. In the event of rectal cancer, either anterior resection or abdominoperineal resection is advised for all *path_MMR* carriers^5,7^. These guideline recommendations are based on the risk of metachronous CRC. A previous Prospective Lynch Syndrome Database (PLSD) report described a 36% cumulative risk of metachronous CRC for *path_MMR* carriers from 40 to 70 years of age after a first CRC^11^. Although numerous retrospective studies and several meta-analyses support the increased risk of metachronous CRC, despite segmental resection, recommendations for extended resection remain a subject of debate due to the current absence of prospective studies and randomized trials. Furthermore, no survival benefit has been demonstrated for extended surgery^12–18^. A quality-of-life comparison between patients who had undergone segmental resection and patients who had undergone subtotal colectomy did not find a significant difference, but the latter group had poorer functional outcomes^19^.

The aim of this study was to prospectively evaluate the risk of metachronous CRC, stratified by gene and the extent of the resection in previous surgery, contributing to the ongoing discussion on surgical strategies for LS patients.

## Methods

### PLSD data

The PLSD background, design, and complete MySQL code are described in a recent publication^20^. The analysis was performed using the latest version of the PLSD data (version 5), thoroughly described in the latest PLSD study^4^. The database contains data from 8500 patients from 25 different countries and provides 76 289 follow-up years, including age at cancer diagnosis, pathology classification, *path_MMR* information, and procedure names for previous and prospectively observed surgery.

The data for this analysis were gathered as previously reported^20,21^. *path_MLH1*, *path_MSH2*, *path_MSH6*, and *path_PMS2* carriers were observed and follow-up years were calculated from an age of 25 years or the age of inclusion until their first prospective CRC, an age of 75 years, or patient death, whichever event came first. *path_PMS2* carriers were not analysed separately due to an insufficient number of carriers and observation years in the database. Follow-up colonoscopies were performed at national expert centres, according to national guidelines presented in a previous publication^22^.

### Annual and cumulative incidences

Annual incidence rates were calculated and are presented for 5-year intervals. Annual and cumulative incidences were determined as outlined in prior publications^4,21^. The confidence intervals for cumulative incidence were calculated using Nelson–Aalen estimates, with an underlying Poisson distribution, as opposed to a normal distribution. This method has been described in detail in previous publications^20,21^. The statistical difference for cumulative incidence was tested using the log rank method. The MySQL code for this analysis was authored by a bioinformatician (Kalle Ojala).

### Surgery annotation

The available surgery data were classified into three categories: minor resection, segmental resection, and extended resection. Minor resection included biopsy, polypectomy, appendectomy, transanal resection, transverse resection, and ileocaecal resection. Segmental resection included right hemicolectomy, extended right hemicolectomy, left hemicolectomy, sigmoid resection for colon cancer, and anterior resection or abdominoperineal resection for rectal cancer. Extended resection included subtotal colectomy with ileosigmoid anastomosis and total abdominal colectomy with ileorectal anastomosis. Patients who had undergone multiple segmental resections before the prospective observation, in which the combined length of the resected colon was equivalent to subtotal or total colectomy, were categorized as part of the extended resection group.

### Survival analysis

Survival analysis was conducted using the Kaplan–Meier method and stratified by surgery type. This analysis was performed separately for two patient groups that partially overlapped: those who underwent surgery before the start of the prospective observation in the study; and those who had the latest surgery after starting the prospective observation. The overall survival was defined as the time from the latest surgical intervention contributing to the categorization (segmental or extended) to death or the end of the prospective observation. All data analyses were performed using R^23^.

## Results

### Patient characteristics

The study sample included 8438 *path_MMR* carriers from 25 countries (*Table 1*). Stratified by gene, there were 3110 (36.9%) *path_MLH1* carriers, 3154 (37.4%) *path_MSH2* carriers, 1634 (19.3%) *path_MSH6* carriers, and 540 (6.4%) *path_PMS2* carriers. The total number of prospective observation years was 65 370, with a mean follow-up time of 7.8 years. The mean age at inclusion for prospective follow-up varied from 43 years for *path_MLH1* carriers to 49 years for *path_PMS2* carriers.

**Table 1:** Patients included, follow-up years, and age at inclusion by gene, history of CRC before inclusion, and country from version 5 of the PLSD

Group	Patients included, number	Follow-up years				Age (years) at inclusion		
		Number	Mean	Min	Max	Mean	Min	Max
All	8438	65 370	7.8	1	40	45	25	73
**Gene**								
*MLH1*	3110	27 900	9.0	1	38	43	25	73
*MSH2*	3154	24 155	7.7	1	31	44	25	73
*MSH6*	1634	10 556	6.5	1	40	48	25	73
*PMS2*	540	2759	5.1	1	23	49	25	73
**History of CRC before inclusion**								
No CRC	5368	43 297	8.1	1	40	42	25	73
CRC	3070	22 073	7.2	1	38	50	25	73
**Country**								
Denmark	1698	15 200	9.0	1	33	44	25	73
Finland	1062	12 800	12.1	1	38	42	25	73
Germany	995	6280	6.3	1	27	44	25	73
Australia	814	6740	8.3	1	33	45	25	73
Spain	693	3450	5.0	1	21	45	25	73
UK	595	3540	6.0	1	36	44	25	73
Holland	523	3460	6.6	1	40	51	25	73
USA	383	2372	6.6	1	16	51	25	73
Italy	326	2380	7.3	1	33	41	25	73
Norway	296	2060	7.0	1	20	44	25	73
Israel	282	1470	5.2	1	31	44	25	73
Canada	180	1280	7.1	1	16	50	25	73
Sweden	153	1350	8.8	1	25	44	25	72
Switzerland	75	448	6.0	1	25	51	28	71
Uruguay	68	460	6.8	1	21	43	25	68
Poland	61	585	9.6	1	22	40	25	69
New Zealand	60	430	7.2	1	12	44	25	67
Brazil	54	421	7.8	1	26	46	25	66
Chile	42	239	5.7	1	12	44	25	66
Argentina	35	205	5.9	1	27	42	27	71
Ireland	18	74	4.1	1	15	48	28	64
Colombia	12	52	4.3	2	7	47	39	60
India	9	19	2.1	1	3	45	33	62
Mexico	3	15	5.0	3	7	28	25	35
Hungary	1	6	6.0	6	6	39	39	39

CRC, colorectal cancer; PLSD, Prospective Lynch Syndrome Database.

At inclusion, 5368 *path_MMR* carriers had not had a prior or prevalent CRC before entering prospective observation, whereas 3070 *path_MMR* carriers had been diagnosed with CRC previously or at first colonoscopy and therefore had prior or prevalent bowel surgery before entering prospective observation. Of these *path_MMR* carriers who had undergone prior colorectal surgery, 2499 (81%) had one CRC, 456 (15%) had two CRCs, 98 had three CRCs, 11 had four CRCs, and 6 had five to six CRCs before entering the study.

First CRC was diagnosed in 489 *path_MMR* carriers and metachronous CRC was diagnosed in 364 *path_MMR* carriers. Staging information was available for 288 metachronous CRCs. Of these, 104 were diagnosed at stage I, 123 were diagnosed at stage II, 53 were diagnosed at stage III, and 8 presented with metastatic disease. The mean(s.d.) time to developing metachronous CRC was 14.3(9.1) years for *path_MLH1* carriers, 12.2(8.0) years for *path_MSH2* carriers, and 10.2(7.4) years for *path_MSH6* carriers.

### Cumulative incidences of primary and metachronous CRC

**Fig. 1:**
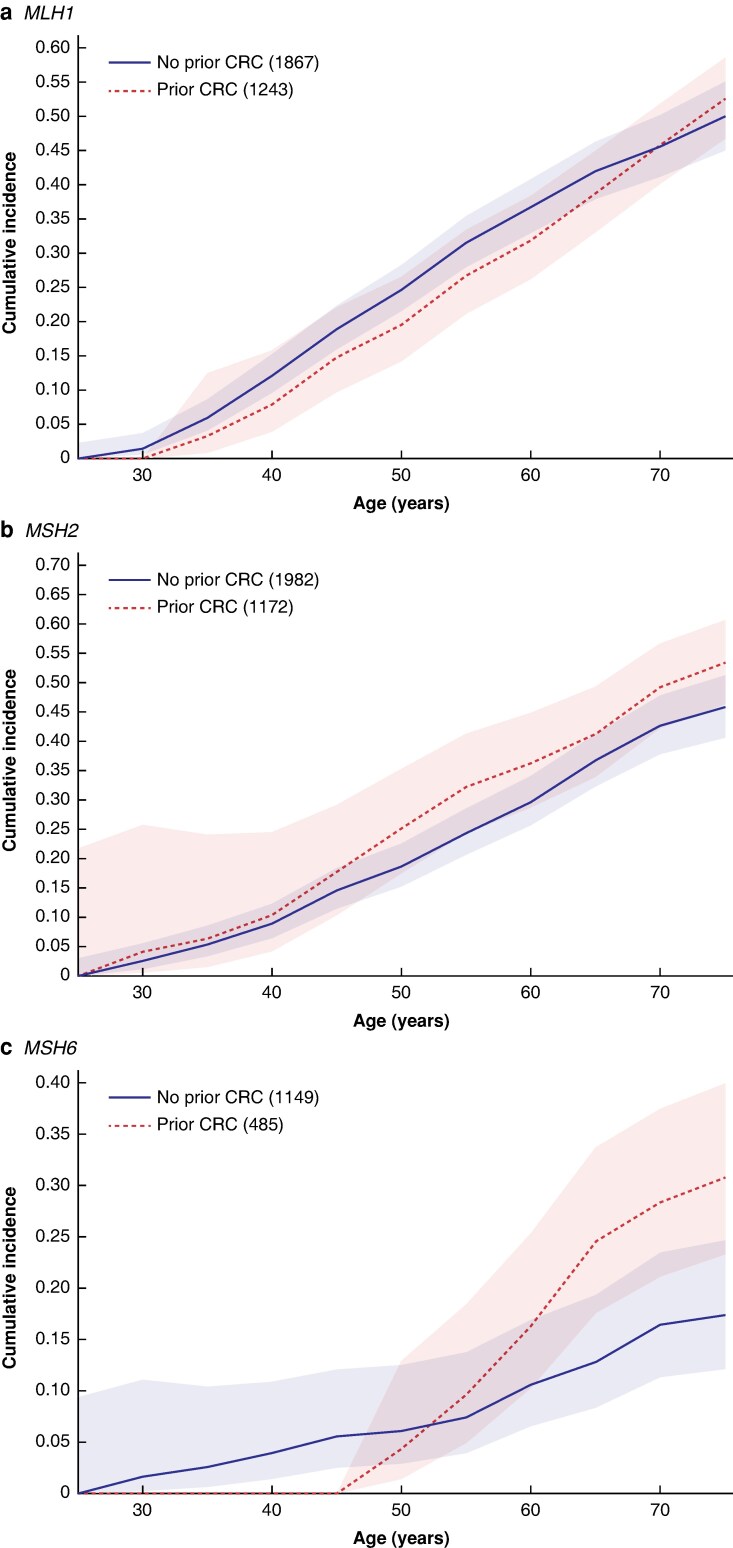
Cumulative incidence of a first CRC for *path_MMR* carriers with no previous CRC and cumulative incidence of metachronous CRC for *path_MMR* carriers with previously diagnosed CRC by gene (with 95% confidence intervals) **a**  *MLH1*. **b**  *MSH2*. **c**  *MSH6*. CRC, colorectal cancer; *path_MMR*, pathogenic MMR variant.

The cumulative incidence of a first CRC by the age of 75 years for *path_MLH1* and *path_MSH2* carriers with no prior CRC was 49.9% (95% c.i. 45.0% to 55.1%) and 45.8% (95% c.i. 40.6% to 51.3%) respectively (*Table 2* and *Fig. 1*). Carriers of *path_MSH6* had a lower cumulative incidence of a first CRC by the age of 75 years (17.4% (95% c.i. 12.1% to 24.7%)).

**Table 2:** Cumulative incidences of first and metachronous CRC by gene

Cancer	Age (years)	Cumulative incidence at age (95% c.i.), %		
		*path_MLH1*	*path_MSH2*	*path_MSH6*
First CRC	30	1.4 (0.5,3.7)	2.5 (1.2,5.6)	1.7 (0.2,11.1)
	40	12.1 (9.6,15.3)	8.9 (6.4,12.4)	4.0 (1.4,10.9)
	50	24.7 (21.4,28.3)	18.6 (15.3,22.7)	6.1 (2.9,12.6)
	60	36.7 (32.9,40.8)	29.6 (25.6,34.1)	10.6 (6.5,17.0)
	70	45.5 (41.2,50.2)	42.7 (37.8,47.8)	16.4 (11.3,23.5)
	75	49.9 (45.0,55.1)	45.8 (40.6,51.3)	17.4 (12.1,24.7)
Metachronous CRC	30	0	4.1 (0.6,25.8)	0
	40	7.9 (3.9,15.8)	10.4 (4.2,24.6)	0
	50	19.5 (14.1,26.6)	25.2 (17.5,35.4)	4.4 (1.4,13.0)
	60	31.9 (26.2,38.4)	36.2 (28.7,45.0)	16.3 (10.3,25.4)
	70	45.8 (40.1,51.9)	49.2 (42.2,56.7)	28.4 (21.1,37.5)
	75	52.5 (46.7,58.5)	53.4 (46.4,60.7)	30.8 (23.3,40.0)

CRC, colorectal cancer; *path_MMR*, pathogenic MMR variant.

For *path_MLH1* carriers, for those who had undergone a bowel resection for CRC previously, the cumulative incidence of metachronous CRC by the age of 75 years was as high as the incidence of a first CRC (52.5% (95% c.i. 46.7% to 58.5%)). For *path_MSH2* carriers with previous CRC the risk of metachronous CRC by the age of 75 years was significantly higher (53.4% (95% c.i. 46.4% to 60.7%)) than the risk of a first CRC (*P* < 0.001). *path_MSH6* carriers with prior or prevalent CRC had a cumulative incidence of metachronous CRC of 30.8% (95% c.i. 23.3% to 40.0%; *P* < 0.001) by the age of 75 years.

### Surgical treatment of CRC diagnosed before inclusion

In total, surgical data contained 112 unique surgery annotations, including synonyms, misspellings, translations, and similar. Surgical procedure history was available for 908 patients (29.6%). Among these, segmental resection was the procedure undertaken in 677 patients (74.6%) and extended resection (or a number of resections equivalent to extended resection) was performed in 155 patients (17.1%) before prospective observation. Minor resection was performed in 22 patients; however, these patients were excluded from the analysis because the procedures did not comply with the principles of oncological surgery. An additional 54 patients were not classified in any of the three categories due to indecipherable annotation (*Table 3*).

**Table 3:** Surgical treatment of CRC before prospective observation, by gene and country

	Segmental resection (*n* = 677)	Extended resection (*n* = 155)
**Gene, *n* (%)**		
*MLH1*	239 (75.2)	79 (24.8)
*MSH2*	231 (78.3)	64 (21.7)
*MSH6*	159 (93.5)	11 (6.5)
*PMS2*	48 (98)	1 (2)
**Country**		
Denmark	306	59
Italy	82	7
Holland	59	2
Finland	54	34
Israel	51	13
UK	37	11
Brazil	21	13
Chile	21	11
Colombia	12	0
Ireland	8	1
Spain	6	2
Switzerland	6	0
India	6	0
USA	3	0
Australia	2	2
Argentina	2	0
Norway	1	0

Values are *n* unless otherwise indicated. CRC, colorectal cancer.

Among *path_MLH1* and *path_MSH2* carriers with CRC, 24.8% and 21.7% underwent extended surgery respectively. Segmental resection was the chosen procedure in 93.5% of the *path_MSH6* carriers and in 48 of 49 of the *path_PMS2* carriers. Right hemicolectomy was the most frequently performed segmental resection.

### Cumulative incidence of metachronous CRC stratified by previous surgical treatment of CRC

**Fig. 2:**
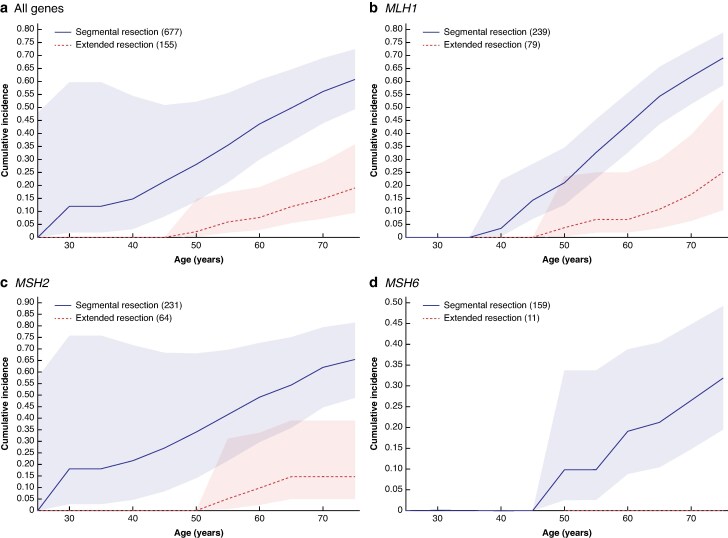
Cumulative incidence of metachronous CRC for *path_MMR* carriers who previously underwent segmental or extended resection by gene (with 95% confidence intervals) **a** All genes. **b**  *MLH1*. **c**  *MSH2*. **d**  *MSH6*. CRC, colorectal cancer; *path_MMR*, pathogenic MMR variant.

The cumulative incidence of metachronous CRC after segmental and extended bowel resections for prior CRC stratified by gene are presented in *Table 4* and illustrated in *Fig. 2*.

**Table 4:** Cumulative incidence of metachronous CRC by previous surgical treatment of CRC and gene

Surgery	Age (years)	Cumulative incidence at age (95% c.i.), %		
		*path_MLH1*	*path_MSH2*	*path_MSH6*
Segmental resection	30	0	18.1 (2.8,75.8)	0
	40	3.5 (0.5,22.5)	21.6 (4.6,71.8)	0
	50	21.1 (12.4,34.6)	34.0 (14.1,68.0)	9.8 (2.6,33.8)
	60	43.4 (32.8,55.7)	49.1 (29.6,72.6)	19.1 (8.7,38.8)
	70	61.8 (51.3,72.4)	62.0 (44.7,79.4)	26.5 (14.8,44.7)
	75	69.1 (58.6,79.0)	65.4 (48.7,81.5)	31.9 (19.5,49.3)
Extended resection	30	0	0	NA
	40	0	0	NA
	50	3.8 (0.5,23.9)	0	0
	60	6.9 (1.8,25.1)	10.0 (2.6,33.8)	0
	70	16.5 (6.3,39.3)	14.7 (5.0,39.0)	0
	75	25.1 (10.4,53.4)	14.7 (5.0,39.0)	0

CRC, colorectal cancer; *path_MMR*, pathogenic MMR variant; NA, not available.

*path_MLH1* carriers had a higher risk of metachronous CRC by the age of 75 years after segmental colorectal resection (69.1% (95% c.i. 58.6% to 79.0%)) compared with extended surgery (25.1% (95% c.i. 10.4% to 53.4%)). Similarly, *path_MSH2* carriers had a cumulative incidence of metachronous CRC of 65.4% (95% c.i. 48.7% to 81.5%) after segmental resection and 14.7% (95% c.i. 5.0% to 39.0%) after extended resection.

The cumulative incidence of metachronous CRC after segmental resection was 31.9% (95% c.i. 19.5% to 49.3%) for *path_MSH6* carriers, whereas no metachronous CRCs were observed in 11 *path_MSH6* carriers who had extended resections (*P* = 0.051).

As a subgroup analysis, the cumulative incidence of metachronous CRC after surgical treatment of previous or prevalent colon cancer was calculated, excluding the previous or prevalent rectal cancers, with no apparent differences to the results (*Fig. S1* and *Table S1*). The cumulative incidence results were also broken down by prospectively observed colon cancer only and prospectively observed rectal cancer only, and also by sex. The rectal cancer rate was unaffected by the extent of the colon resections, whereas the extended colon resections reduced the risk of metachronous colon cancers in both males and females (*Figs S2–S4*). The cumulative incidence of metachronous CRC was analysed based on the location of prior segmental resection (right *versus* left), with no significant difference observed (*Fig. S5*).

### Survival

Survival data after CRC were available for 688 (8.2*%) path_MMR* carriers. Of these, 169 patients underwent either segmental or extended resection; surgical data were not available for the remaining 519 patients.

**Fig. 3:**
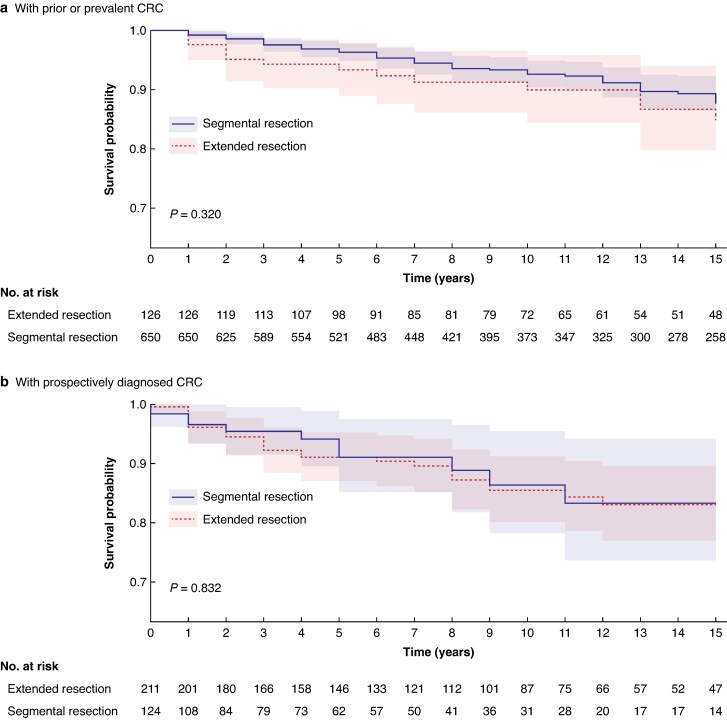
Overall survival of *path_MM*R carriers for segmental resection and extended colectomy groups **a** With prior or prevalent CRC. **b** With prospectively diagnosed CRC. CRC, colorectal cancer; *path_MMR*, pathogenic MMR variant.

No difference in overall survival 15 years after the last surgery was observed between the segmental resection and extended colectomy cohorts within the group that had surgery before entering prospective observation (*P* = 0.320) or the group that had surgery after entering prospective observation (*P* = 0.832) (*Fig. 3*).

## Discussion

This large prospective observational study compares the incidence of metachronous CRC after partial *versus* extended surgical resection for CRC in patients with LS. It demonstrated that, for *path_MLH1*, *path_MSH2*, and *path_MSH6* carriers, the risk of metachronous CRC was lower in those having extended compared with segmental resection, despite continued endoscopic surveillance. LS patients with previous standard segmental resections for CRC had a similar or higher prospectively observed cumulative incidence of metachronous CRC compared with the corresponding incidence of a first CRC for *path_MMR* carriers who had no previous CRC. When stratified by gene, the confidence intervals were wide, but those with previous CRC appeared to have an elevated risk of metachronous CRC compared with the risk of a first CRC in those without previous CRC. This challenges the conclusion made in the 2017 PLSD paper that was based on a smaller cohort and did not observe this increase in risk^11^.

This study included confirmed class 4 or 5^24^  *path_MMR* carriers with no previous CRC history, as well as *path_MMR* carriers who had survived primary CRC after undergoing segmental or extended large bowel resection, thereby allowing for the calculations of cumulative risks of first and metachronous CRCs. LS patients with prior CRC had a risk of metachronous CRC that was similar to the risk of a first CRC for *path_MLH1* carriers and a risk of metachronous CRC that was higher than the risk of a first CRC for *path_MSH2* and *path_MSH6* carriers. However, this direct comparison ignores the fact that many previously identified *path_MMR* carriers undergo extended resection for their CRC and the residual risk after any resection should be lower than when the whole bowel remains *in situ*. This suggests that certain additional risk-increasing factors contribute to individual CRC risk. The lack of reduction in metachronous cancer risk compared with the risk of a first CRC should be weighed against the type of surgical resection and also raises the question of whether shorter colonoscopic surveillance intervals should be used for those with previous CRC compared with those without previous CRC.

A recent retrospective study from the Netherlands reported an incidence rate of 16% for metachronous CRC after segmental resection for *path_MSH6* and *path_PMS2* carriers^25^. Interestingly, in the present study, the cumulative incidence of prospectively observed metachronous CRC after standard segmental resection for the first CRC was substantially higher than the cumulative incidence of a first CRC for *path_MSH6* carriers (*Fig. 1c*). Extended resections were undertaken rarely for *path_MSH6* carriers. However, none of the *path_MSH6* carriers with available data who underwent extended resection in the present study developed metachronous CRC. Those with previous CRC and *path_MSH6* may have additional risk factors that increase their incidence of CRC. Another possible explanation is that those with previous CRC have already reached the point where the immune system is less effective in controlling MMR-deficient carcinogenesis and previous CRC serves as a surrogate for selecting out those with increased risk. Nevertheless, the observation of an increased risk of metachronous CRC in those who had standard resections followed by surveillance, compared with those with no previous CRC undergoing surveillance, leads the authors to believe that extended colectomy significantly reduces the risk of metachronous CRC for *path_MSH6* carriers. This suggests that surgical recommendations for *path_MSH6* carriers with CRC that currently favour segmental resection should be revisited^5^.

A previous PLSD study found the cumulative risk of primary CRC for *path_PMS2* carriers at the age of 75 years is 10.4% (95% c.i. 2.9% to 40.8%)^26^. Similar results were also reported by ten Broeke *et al*.^27^. Another previous PLSD study attempted to calculate the risk of metachronous CRC for *path_PMS2* carriers, but was unsuccessful due to a limited number of patients (47 patients)^11^. Although the present study had a multicentre design and a larger sample size (540 patients), the low number of metachronous CRC events did not allow reliable calculations of the cumulative incidence of subsequent CRC for *path_PMS2* carriers. The limited findings of low metachronous CRC risk support the current guideline recommendations, favouring segmental resection for the treatment of a first CRC for *path_PMS2* carriers.

Although guidelines recommend extended large bowel resection for the first CRC for *path_MLH1* and *path_MSH2* carriers, segmental resection was performed in the majority of such patients^5–8^. Based on the breakdown of data by the colon and rectum in the present study, it seems that only occasional sigmoid colon cancers appear after subtotal colectomy, whereas the rate of rectal cancer is unchanged whether or not the colon is removed. The proportion of patients receiving confirmation of their LS diagnoses after their first CRC may, in part, account for the notable frequency of segmental resections observed and this may vary by country. However, if LS is confirmed before treating a first or metachronous CRC, it is imperative to provide updated information to patients. This should include discussion about the risk of metachronous CRC, potential surgical complications, CRC surveillance after surgery, and expected quality-of-life and functional outcomes associated with both segmental and extended bowel resection. The choice of surgical approach should therefore be tailored accordingly.

The most recent meta-analysis by Toh *et al*.^28^ indicates that LS carriers diagnosed with primary CRC have a 5-year overall survival rate of 90% and a 10-year overall survival rate of 80%. However, a survival advantage for extended resection compared with segmental resection has not been demonstrated previously^12–18^. The present study did not find any survival benefit either. The lack of an observed survival benefit may be attributed to several factors. LS patients typically undergo regular colonoscopies every 2–3 years, facilitating diagnosis at an early stage, and LS-associated CRCs are generally associated with a better prognosis. Dominguez-Valentin *et al*.^29^ analysed LS patients under colonoscopic surveillance who developed their first colon cancer, and 81% were diagnosed with stage I or II colon cancer; the 10-year survival after colon cancer was 93%, 94%, and 82% for stage I, stage II, and stage III disease respectively, much higher than for sporadic cancer. Advances in surgical techniques and the prompt detection of complications through CT imaging have also contributed to improved survival outcomes after colorectal surgery. Given that survival rates do not differ significantly with extended surgery, it is essential to discuss both surgical options with patients, considering their personal preferences and lifestyles, to determine the most suitable treatment.

A limitation of the present study is the under-reporting of the type of surgery and survival data to the PLSD. The contributing centres reporting the type of surgery may have been more aware of the surgical guidelines and performed extended bowel resections in LS carriers more often than the centres that did not record the type of surgery. The strengths of the study are the prospective design and the number of extended colectomies that was large enough to statistically compare outcomes between segmental and extended colectomies, stratified by the MMR gene involved.

This study has found that *path_MLH1* and *path_MSH2* carriers who undergo segmental resection for a first CRC were at increased risk of metachronous CRC compared with those with no prior CRC. This increased risk also applied to *path_MSH6* carriers, suggesting extended surgery may also be indicated to manage their first CRC. Extended resection for LS-associated CRC substantially decreased the risk of metachronous CRC compared with segmental resection in all three groups of *path_MMR* carriers.

## Collaborators

K. Zalevskaja (Applied Tumor Genomics Research Program, Research Programs Unit, University of Helsinki, Helsinki, Finland; Department of Surgery, Mikkeli Central Hospital, Mikkeli, Finland); K. Ojala (Applied Tumor Genomics Research Program, Research Programs Unit, University of Helsinki, Helsinki, Finland; Department of Abdominal Surgery, Helsinki University Hospital, Helsinki, Finland); A. Petrov (Faculty of Medicine and Health Technology, Tampere University and Tays Cancer Centre, Tampere University Hospital, Tampere, Finland); S. Haupt (Engineering Mathematics and Computing Lab (EMCL), Interdisciplinary Center for Scientific Computing (IWR), Heidelberg University, Heidelberg, Germany; Data Mining and Uncertainty Quantification (DMQ), Heidelberg Institute for Theoretical Studies (HITS), Heidelberg, Germany); L. Sunde (Department of Clinical Genetics, Aalborg University Hospital, Aalborg, Denmark; Department of Clinical Medicine, Aalborg University, Gistrup, Denmark); I. Bernstein (Department of Quality and Coherence, Aalborg University Hospital, Aalborg University, Aalborg, Denmark); M. A. Jenkins (Melbourne School of Population and Global Health, Centre for Epidemiology and Biostatistics, The University of Melbourne, Parkville, Australia); S. Aretz (Institute of Human Genetics, National Center for Hereditary Tumor Syndromes, Medical Faculty, University Hospital Bonn, University of Bonn, Bonn, Germany); M. Nielsen (Department of Clinical Genetics, Leids Universitair Medisch Centrum, Leiden, The Netherlands); G. Capella (Hereditary Cancer Program, Institut Català d'Oncologia-IDIBELL, Barcelona, Spain); F. Balaguer (Gastroenterology Department, Hospital Clínic de Barcelona, Barcelona, Spain); D. G. Evans (Manchester Centre for Genomic Medicine, Manchester University NHS Foundation Trust, Manchester, UK); J. Burn (Faculty of Medical Sciences, Newcastle University, Newcastle Upon Tyne, UK); E. Holinski-Feder (Campus Innenstadt, Medizinische Klinik und Poliklinik IV, Klinikum der Universität München, Munich, Germany; Center of Medical Genetics, Munich, Germany); L. Bertario (Division of Cancer Prevention and Genetics, European Institute of Oncology IRCCS, Milan, Italy); B. Bonanni (Division of Cancer Prevention and Genetics, European Institute of Oncology IRCCS, Milan, Italy); A. Lindblom (Department of Molecular Medicine and Surgery, Karolinska Institutet, Stockholm, Sweden); Z. Levi (Service High Risk GI Cancer Gastroenterology, Department Rabin Medical Center, Israel); F. Macrae (Colorectal Medicine and Genetics, The Royal Melbourne Hospital, Melbourne, Australia; Department of Medicine, Melbourne University, Melbourne, Australia); I. Winship (Colorectal Medicine and Genetics, The Royal Melbourne Hospital, Melbourne, Australia; Department of Medicine, Melbourne University, Melbourne, Australia; Department of Medicine, University of Melbourne, Melbourne, Australia); J.-P. Plazzer (Colorectal Medicine and Genetics, The Royal Melbourne Hospital, Melbourne, Australia; Department of Medicine, Melbourne University, Melbourne, Australia); R. Sijmons (Department of Genetics, University of Groningen, University Medical Center Groningen, Groningen, The Netherlands); L. Laghi (Laboratory of Molecular Gastroenterology, IRCCS Humanitas Research Hospital, Rozzano, Italy; Department of Medicine and Surgery, University of Parma, Parma, Italy); A. Della Valle (Hospital Fuerzas Armadas, Grupo Colaborativo Uruguayo, Investigación de Afecciones Oncológicas Hereditarias, Montevideo, Uruguay); K. Heinimann (Medical Genetics, Institute for Medical Genetics and Pathology, University Hospital Basel, Basel, Switzerland); T. Dębniak (Department of Genetics and Pathology, International Hereditary Cancer Center, Szczecin, Poland); R. Fruscio (Department of Medicine and Surgery, University of Milan Bicocca, A.O. San Gerardo, Clinic of Obstetrics and Gynecology, Monza, Italy); F. Lopez-Koestner (Clínica Universidad de los Andes, Santiago, Chile; Programa Cáncer Heredo Familiar, Santiago, Chile); K. Alvarez-Valenzuela (Clínica Universidad de los Andes, Santiago, Chile; Programa Cáncer Heredo Familiar, Santiago, Chile); L. H. Katz (Department of Gastroenterology, Hadassah Medical Center, Faculty of Medicine, Hebrew University of Jerusalem, Israel); I. Laish (Department of Gastroenterology, Hadassah Medical Center, Faculty of Medicine, Hebrew University of Jerusalem, Israel); E. Vainer (Hadassah Medical Center, Israel); C. Vaccaro (Hereditary Cancer Program (PROCANHE), Hospital Italiano de Buenos Aires, Buenos Aires, Argentina); D. M. Carraro (Clinical and Functional Genomics Group, A.C.Camargo Cancer Center, Sao Paulo, Brazil); K. Monahan (Lynch Syndrome & Family Cancer Clinic, St Mark's Hospital, London, UK); E. Half (Gastrointestinal Cancer Prevention Unit, Gastroenterology Department, Rambam Health Care Campus, Haifa, Israel); A. Stakelum (St Vincent's University Hospital, Ireland); D. Winter (St Vincent's University Hospital, Ireland); R. Kennelly (St Vincent's University Hospital, Ireland); N. Gluck (Department of Gastroenterology, Tel-Aviv Sourasky Medical Center and Sackler Faculty of Medicine, Tel-Aviv University, Tel-Aviv, Israel); H. Sheth (Foundation for Research in Genetics and Endocrinology, Institute of Human Genetics, Ahmedabad, India); N. Abu-Freha (Soroka University Medical Center, Ben-Gurion University of the Negev, Beer Sheva, Israel); M. Greenblatt (University of Vermont, Larner College of Medicine, Burlington, VT, USA); B. M. Rossi (Hospital Sirio Libanes, Sao Paulo, Brazil); M. Bohorquez (University of Tolima, Tolima, Colombia); G. M. Cavestro (Gastroenterology and Gastrointestinal Endoscopy Unit, Division of Experimental Oncology, IRCCS San Raffaele Scientific Institute, Vita-Salute San Raffaele University, Milan, Italy); L. S. Lino-Silva (Surgical Pathology, Instituto Nacional de Cancerologia, Mexico City, Mexico); K. Horisberger (Department of Surgery, Universitätsmedizin Mainz, Mainz, Germany); M. G. Tibiletti (Ospedale di Circolo ASST Settelaghi, Centro di Ricerca tumori eredo-familiari, Università dell'Insubria, Varese, Italy); I. do Nascimento (Universidade Federal de Bahia, Bahia, Brazil); H. Thomas (St Mark's Hospital, Department of Surgery and Cancer, Imperial College London, London, UK); N. T. Rossi (Fundación para el Progreso de la Medicina, Sanatorio Allende, Córdoba, Argentina); L. A. da Silva (Hospital Universitário Oswaldo Cruz, Universidade de Pernambuco, Recife, Brazil; SEQUIPE, Recife, Brazil); A. Zaránd (1st Department of Surgery, Semmelweis University, Budapest, Hungary); J. Ruiz-Bañobre (Department of Medical Oncology, University Clinical Hospital of Santiago de Compostela, Santiago de Compostela, Spain; Centro de Investigación Biomédica en Red Cáncer (CIBERONC), Madrid, Spain); V. Heuveline (Engineering Mathematics and Computing Lab (EMCL), Interdisciplinary Center for Scientific Computing (IWR), Heidelberg University, Heidelberg, Germany; Data Mining and Uncertainty Quantification (DMQ), Heidelberg Institute for Theoretical Studies (HITS), Heidelberg, Germany); L. J. Lindberg (The Danish HNPCC Register, Gastrounit, Copenhagen University Hospital-Amager and Hvidovre Hospital, Hvidovre, Denmark); I. Gögenur (Department of Surgery, Center for Surgical Science, Zealand University Hospital, Denmark; Institute for Clinical Medicine, Copenhagen University, Denmark); J. L. Hopper (Centre for Epidemiology and Biostatistics, Melbourne School of Population and Global Health, The University of Melbourne, Parkville, Australia); A. K. Win (Centre for Epidemiology and Biostatistics, Melbourne School of Population and Global Health, The University of Melbourne, Parkville, Australia); R. W. Haile (Department of Medicine, Division of Oncology, Stanford Cancer Institute, Stanford University, Stanford, CA, USA); N. Lindor (Department of Health Science Research, Mayo Clinic Arizona, USA); S. Gallinger (Lunenfeld Tanenbaum Research Institute, Mount Sinai Hospital, University of Toronto, Toronto, Canada); L. Le Marchand (University of Hawaii Cancer Center, Honolulu, HI, USA); P. A. Newcomb (Public Health Sciences Division, Fred Hutchinson Cancer Research Center, Seattle, WA, USA); J. Figueiredo (Public Health Sciences Division, Fred Hutchinson Cancer Research Center, Seattle, WA, USA); D. D. Buchanan (Colorectal Oncogenomics Group, Department of Clinical Pathology, The University of Melbourne, Parkville, Australia; University of Melbourne Centre for Cancer Research, Victorian Comprehensive Cancer Centre, Parkville, Australia; Genomic Medicine and Family Cancer Clinic, Royal Melbourne Hospital, Parkville, Australia); S. N. Thibodeau (Department of Laboratory Medicine and Pathology, Mayo Clinic, Rochester, NY, USA); M. v. Knebel Doeberitz (Department of Applied Tumour Biology, Institute of Pathology, University Hospital Heidelberg, Heidelberg, Germany; Cooperation Unit Applied Tumour Biology, German Cancer Research Center (DKFZ), Heidelberg, Germany); M. Loeffler (Institute for Medical Informatics, Statistics and Epidemiology, University of Leipzig, Leipzig, Germany); N. Rahner (Institute of Human Genetics, Medical Faculty and University Hospital Düsseldorf, Heinrich-Heine-University Düsseldorf, Germany); E. Schröck (National Center for Tumor Diseases (NCT), Partner Site Dresden, Dresden, Germany; German Cancer Consortium (DKTK) Dresden and German Cancer Research Center (DKFZ) Heidelberg, Heidelberg, Germany; Institute for Clinical Genetics, Faculty of Medicine and University Hospital Carl Gustav Carus, TU Dresden, Dresden, Germany; Hereditary Cancer Syndrome Center Dresden, Faculty of Medicine and University Hospital Carl Gustav Carus, TU Dresden, Dresden, Germany); V. Steinke-Lange (Medizinische Klinik und Poliklinik IV, Campus Innenstadt, Klinikum der Universität München, Munich, Germany; MGZ-Medical Genetics Center, Munich, Germany); W. Schmiegel (Department of Medicine, Knappschaftskrankenhaus, Ruhr-University Bochum, Bochum, Germany); D. Vangala (Department of Medicine, Knappschaftskrankenhaus, Ruhr-University Bochum, Bochum, Germany); C. Perne (Institute of Human Genetics, National Center for Hereditary Tumor Syndromes, Medical Faculty, University Hospital Bonn, University of Bonn, Bonn, Germany); R. Hüneburg (Department of Internal Medicine, University Hospital Bonn, Bonn, Germany); S. Redler (German Cancer Consortium (DKTK) Dresden and German Cancer Research Center (DKFZ) Heidelberg, Heidelberg, Germany); R. Büttner (Institute of Pathology, Faculty of Medicine and University Hospital Cologne, Cologne, Germany); J. Weitz (Technische Universität Dresden, Dresden, Germany); M. Pineda (Hereditary Cancer Program, Institut Català d'Oncologia-IDIBELL, Barcelona, Spain); N. Duenas (Hereditary Cancer Program, Institut Català d'Oncologia-IDIBELL, Barcelona, Spain); J. Brunet Vidal (Hereditary Cancer Program, Institut Català d'Oncologia-IDIBELL, Barcelona, Spain); L. Moreira (Gastroenterology Department, Hospital Clínic de Barcelona, Barcelona, Spain); A. Sánchez (Gastroenterology Department, Hospital Clínic de Barcelona, Barcelona, Spain); J. Castillo-Iturra (Gastroenterology Department, Hospital Clínic de Barcelona, Barcelona, Spain); E. Hovig (Department of Tumor Biology, Institute of Cancer Research, The Norwegian Radium Hospital, Oslo, Norway; Centre for Bioinformatics, Department of Informatics, University of Oslo, Oslo, Norway); K. Green (Manchester Centre for Genomic Medicine, Manchester University NHS Foundation Trust, Manchester, UK); F. Lalloo (Manchester Centre for Genomic Medicine, Manchester University NHS Foundation Trust, Manchester, UK); J. Hill (Department of Surgery, Central Manchester University Hospitals NHS Foundation Trust and University of Manchester, Manchester, UK); E. Crosbie (Gynaecological Oncology Research Group, Manchester University NHS Foundation Trust, Manchester, UK; Division of Cancer Sciences, University of Manchester, Manchester, UK); M. Mints (Division of Obstetrics and Gynaecology, Department of Women's and Children's Health, Karolinska Institutet, Karolinska University Hospital, Stockholm, Sweden); Y. Goldberg (Head Adult Genetic Service, Raphael Recanati Genetic Institute, Rabin Medical Center-Beilinson Hospital, Petach Tikva, Israel); D. Tjandra (Colorectal Medicine and Genetics, The Royal Melbourne Hospital, Melbourne, Australia; Department of Medicine, Melbourne University, Melbourne, Australia); S. W. ten Broeke (Department of Genetics, University of Groningen, University Medical Center Groningen, Groningen, The Netherlands); R. Kariv (St Vincent's University Hospital, Ireland); G. Rosner (St Vincent's University Hospital, Ireland); A. Jain (Gastrol Hospital, Ahmedabad, India); P. Shah (Zydus Cancer Centre, Ahmedabad, India); M. Shah (Zydus Cancer Centre, Ahmedabad, India); F. Neffa (Laboratory of Molecular Gastroenterology, IRCCS Humanitas Research Hospital, Rozzano, Italy; Department of Medicine and Surgery, University of Parma, Parma, Italy); P. Esperon (Laboratory of Molecular Gastroenterology, IRCCS Humanitas Research Hospital, Rozzano, Italy; Department of Medicine and Surgery, University of Parma, Parma, Italy); W. Pavicic (Instituto de Medicina Traslacional e Ingenieria Biomedica (IMTIB), CONICET IU, Hospital Italiano de Buenos Aires, Buenos Aires, Argentina); G. T. Torrezan (Clinical and Functional Genomics Group, A.C. Camargo Cancer Center, Sao Paulo, Brazil); T. Bassaneze (University of Vermont, Larner College of Medicine, Burlington, VT, USA); C. A. Martin (Hospital Privado Universiatrio de Córdoba, Cordoba, Argentina); K. Pylvänäinen (Department of Education and Science, The Wellbeing Services of Central Finland, Jyväskylä, Finland); G. Möslein (Surgical Center for Hereditary Tumors, Ev. Bethesda Khs Duisburg, University Witten-Herdecke, Herdecke, Germany); A. Lepistö (Applied Tumor Genomics Research Program, Research Programs Unit, University of Helsinki, Helsinki, Finland; Department of Abdominal Surgery, Helsinki University Hospital, Helsinki, Finland); J.-P. Mecklin (Faculty of Sport and Health Sciences, University of Jyväskylä, Jyväskylä, Finland; Department of Education and Research, The Wellbeing Services of Central Finland, Jyväskylä, Finland); L. Renkonen-Sinisalo (Applied Tumor Genomics Research Program, Research Programs Unit, University of Helsinki, Helsinki, Finland; Department of Abdominal Surgery, Helsinki University Hospital, Helsinki, Finland); J. R. Sampson (Division of Cancer and Genetics, Cardiff University School of Medicine, Cardiff, UK); M. Dominguez Valentin (Department of Tumor Biology, Institute of Cancer Research, The Norwegian Radium Hospital, Oslo, Norway); P. Møller (Department of Tumor Biology, Institute of Cancer Research, The Norwegian Radium Hospital, Oslo, Norway); T. T. Seppälä (Applied Tumor Genomics Research Program, Research Programs Unit, University of Helsinki, Helsinki, Finland; Department of Abdominal Surgery, Helsinki University Hospital, Helsinki, Finland; Faculty of Medicine and Health Technology, Tampere University and Tays Cancer Centre, Tampere University Hospital, Tampere, Finland).

## Supplementary Material

znaf061_Supplementary_Data

## Data Availability

Individual patient data are not publicly available due to restrictions with regard to data privacy. Aggregate results based on publications can be viewed at www.PLSD.eu.
