# Supplementary material for: Metachronous colorectal cancer risks after extended or segmental resection in MLH1, MSH2, and MSH6 Lynch syndrome: multicentre study from the Prospective Lynch Syndrome Database
Source: Br J Surg. 2025 Apr 15;112(4):znaf061. doi: 10.1093/bjs/znaf061 (PMC11997434; doi:10.1093/bjs/znaf061)
Supplement: znaf061_Supplementary_Data [file znaf061_supplementary_data.docx]

**Metachronous colorectal cancer risks after extended or segmental resection in *MLH1-, MSH2*- and *MSH6* Lynch syndrome: a multi-centre study from the Prospective Lynch Syndrome Database**

Authors

*Prospective Lynch Syndrome Database*

**Corresponding author**

Dr. Kristina Zalevskaja

Department of Surgery, Porrassalmenkatu 35-37, 50100, Mikkeli, Finland

**ORCID ID** 0009-0008-6437-1448; **Twitter** @k_zalevskaja

**Supplementary Materials - Index**

| **Supplementary Figures and Tables** |  |
| --- | --- |
| Table S1. Cumulative incidence of metachronous CRC by surgical treatment of prior or prevalent colon cancer by gene | *page 2* |
| Figure S1. Cumulative incidence of metachronous CRC for *path_MMR* carriers that previously underwent segmental (blue) or extended (red) resection for prior or prevalent colon cancer by gene (with 95% CIs). | *page 3* |
| Figure S2. Cumulative incidence of metachronous CRC for *path_MMR* carriers that previously underwent segmental (blue) or extended (red) resection for prior or prevalent CRC by gene and gender (with 95% CIs) | *page 5* |
| Figure S3. Cumulative incidence of metachronous colon cancer for *path_MMR* carriers that previously underwent segmental (blue) or extended (red) resection for prior or prevalent CRC by gene and gender (with 95% CIs) | *page 6* |
| Figure S4. Cumulative incidence of metachronous rectal cancer for *path_MMR* carriers that previously underwent segmental (blue) or extended (red) resection for prior or prevalent CRC by gene and gender (with 95% CIs) | *page 7* |
| Figure S5. Cumulative incidence of metachronous CRC for *path_MMR* carriers that underwent right hemicolectomy (red) or left hemicolectomy (blue) for prior or prevalent CRC | *page 8* |

**Supplementary Figures and Tables**

**Table S1** Cumulative incidence (with 95% CI) of metachronous CRC by surgical treatment of previous or prevalent colon cancer by gene

| **Surgery** | **Age** | **Cumulative incidence at age (% (95% CI))** | | |
| --- | --- | --- | --- | --- |
|  |  | ***path_MLH1*** | ***path_MSH2*** | ***path_MSH6*** |
| Segmental resection | 30 | 0 | 30.0 (4.9 – 92.1) | 0 |
|  | 40 | 3.7 (0.5 – 23.3) | 34.1 (7.3 – 89.9) | 0 |
|  | 50 | 22.4 (13.2 – 36.4) | 43.9 (15.3 – 86.7) | 9.0 (1.3 – 48.8) |
|  | 60 | 41.7 (30.8 – 54.7) | 57.3 (30.0 – 86.8) | 18.0 (6.4 – 45.1) |
|  | 70 | 60.3 (49.3 – 71.5) | 67.1 (43.0 – 88.9) | 27.0 (13.4 – 49.9) |
|  | 75 | 68.1 (57.1 – 78.6) | 68.9 (45.5 – 89.5) | 33.2 (18.8 – 54.2) |
|  |  |  |  |  |
| Extended resection | 30 | 0 | 0 | *NA* |
|  | 40 | 0 | 0 | *NA* |
|  | 50 | 3.9 (0.6 – 24.7) | 0 | 0 |
|  | 60 | 7.3 (1.9 – 26.2) | 9.8 (2.6 – 33.8) | 0 |
|  | 70 | 16.9 (6.5 – 39.8) | 14.7 (5.0 – 39.3) | 0 |
|  | 75 | 25.4 (10.6 – 53.7) | 13.5 (5.0 – 39.3) | 0 |
| *NA*- not available |  |  |  |  |

**Figure S1** Cumulative incidence of **metachronous CRC** for *path_MMR* carriers that previously underwent segmental (blue) or extended (red) resection for **prior or prevalent** **colon cancer** by gene (with 95% CIs). **a** all genes, **b** *MLH1*, **c** *MSH2*, **d** *MSH6*.

**a** all genes


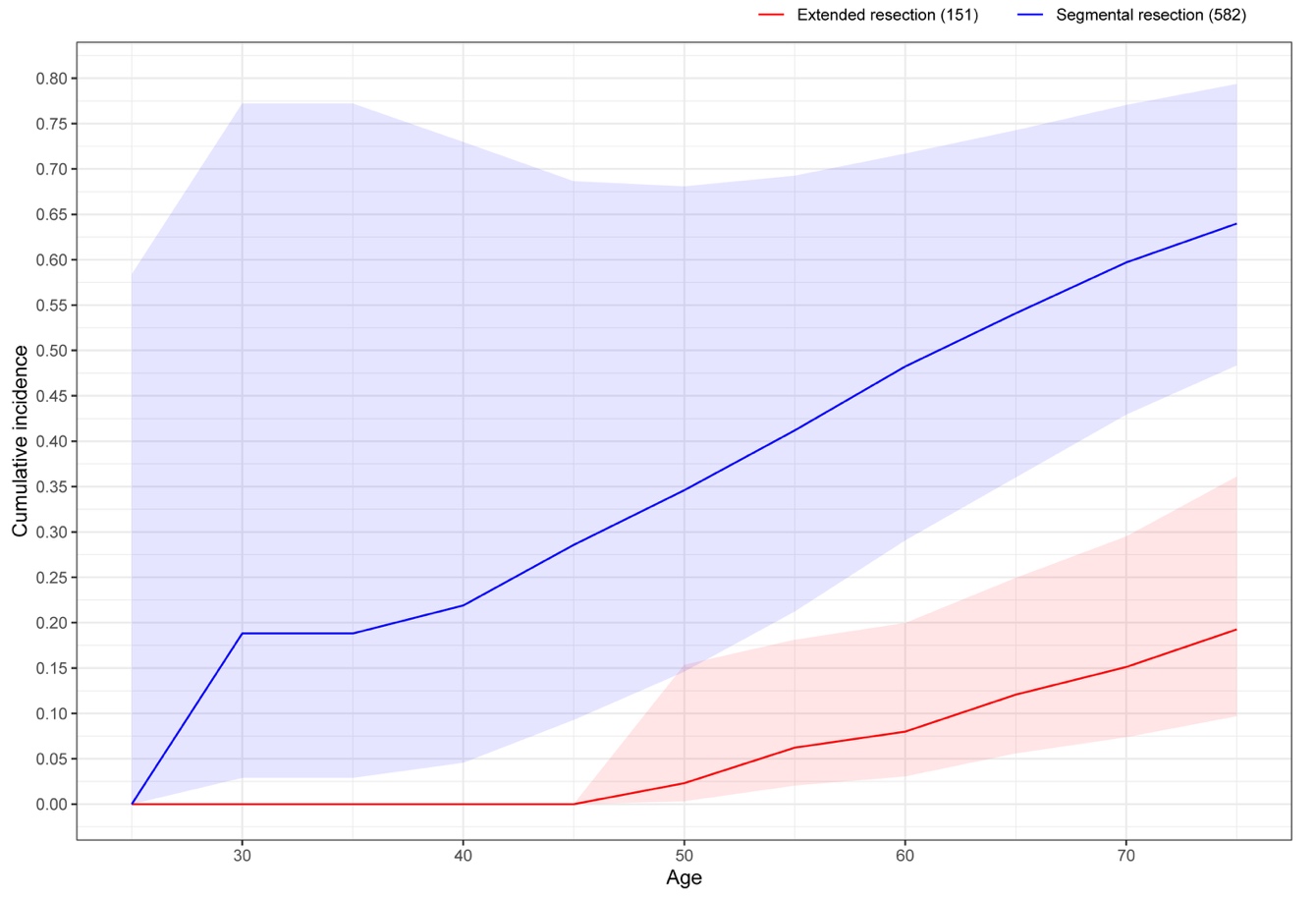


**b** *MLH1*


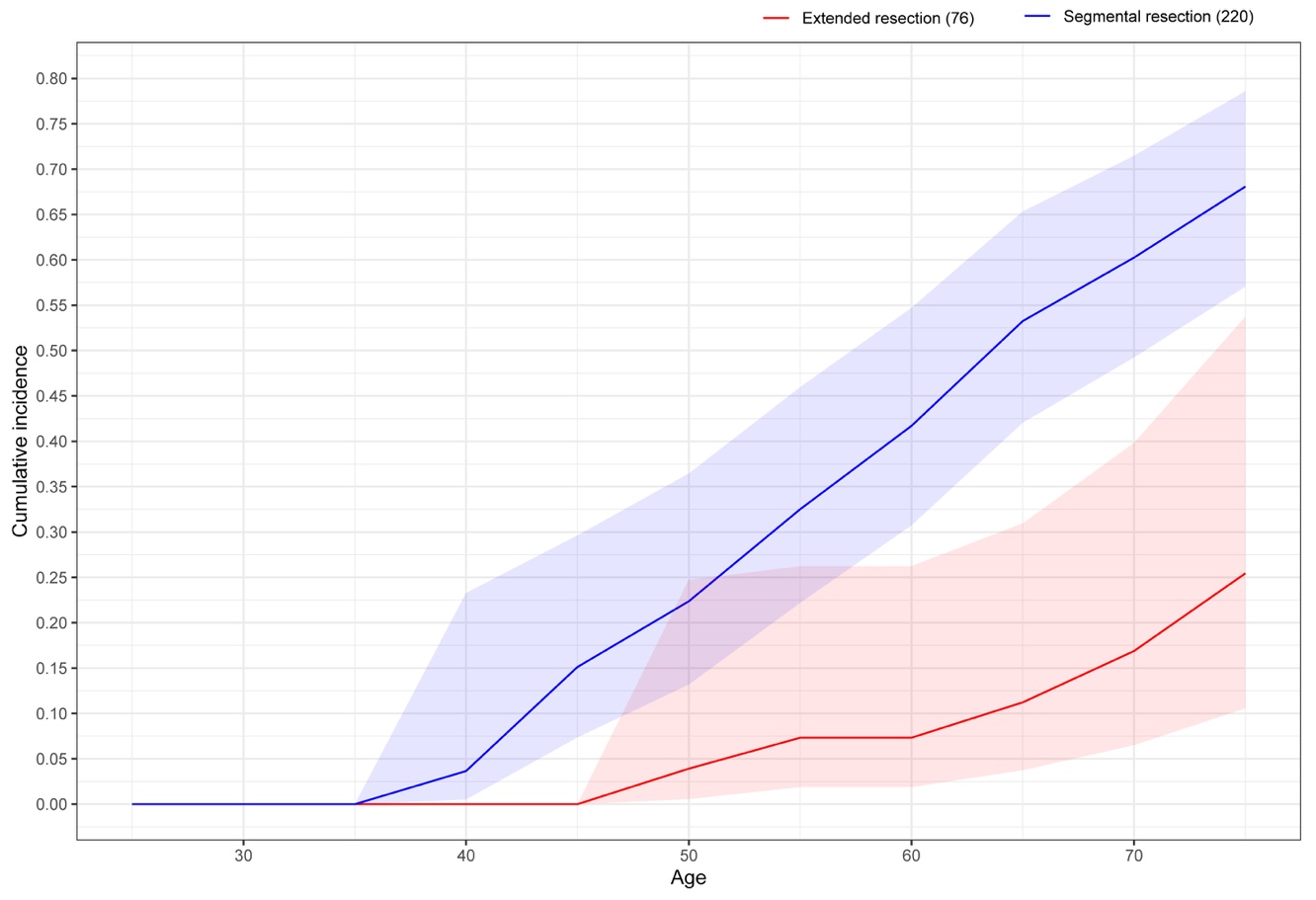


**c** *MSH2*

*
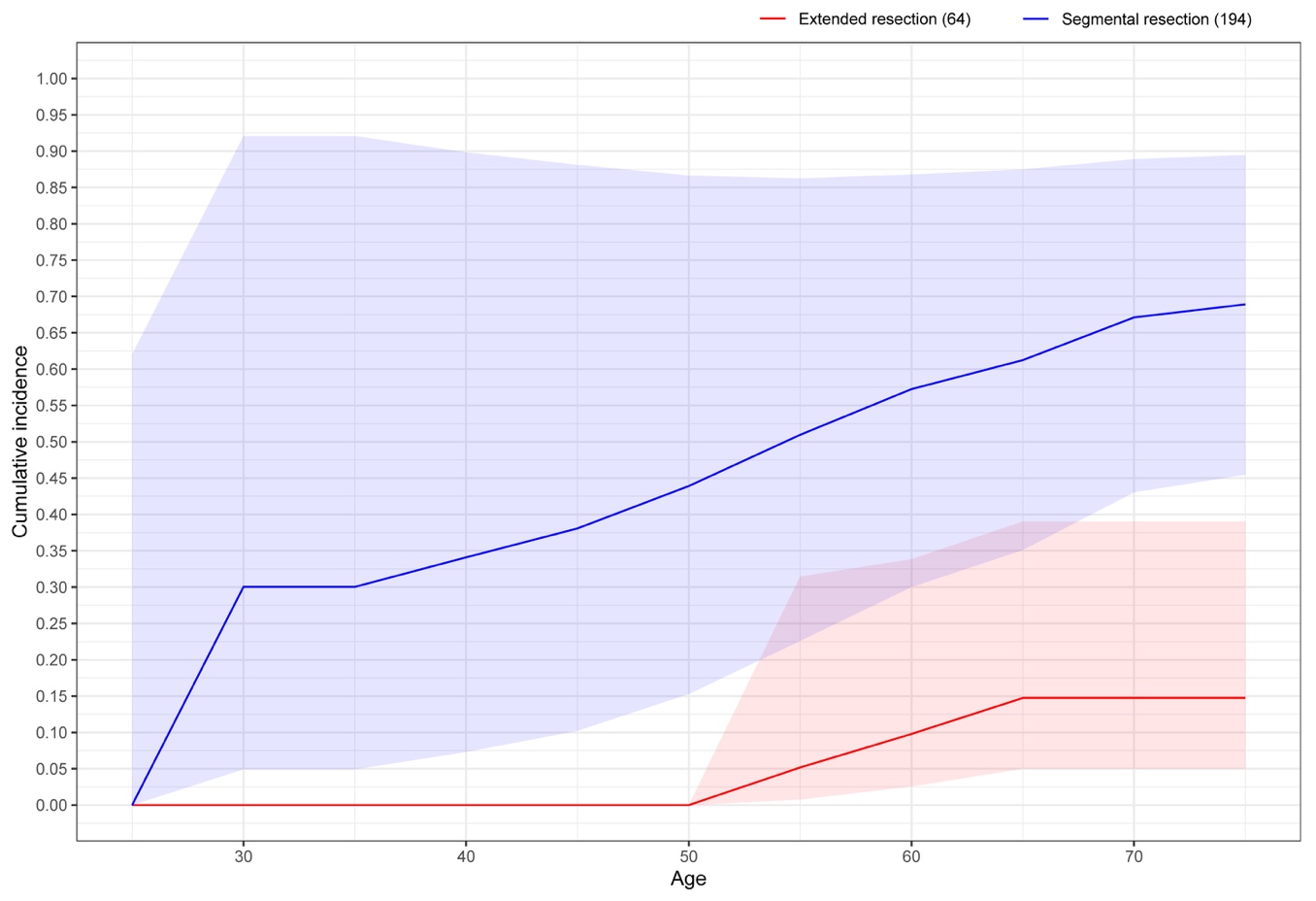
*

**d** *MSH6*


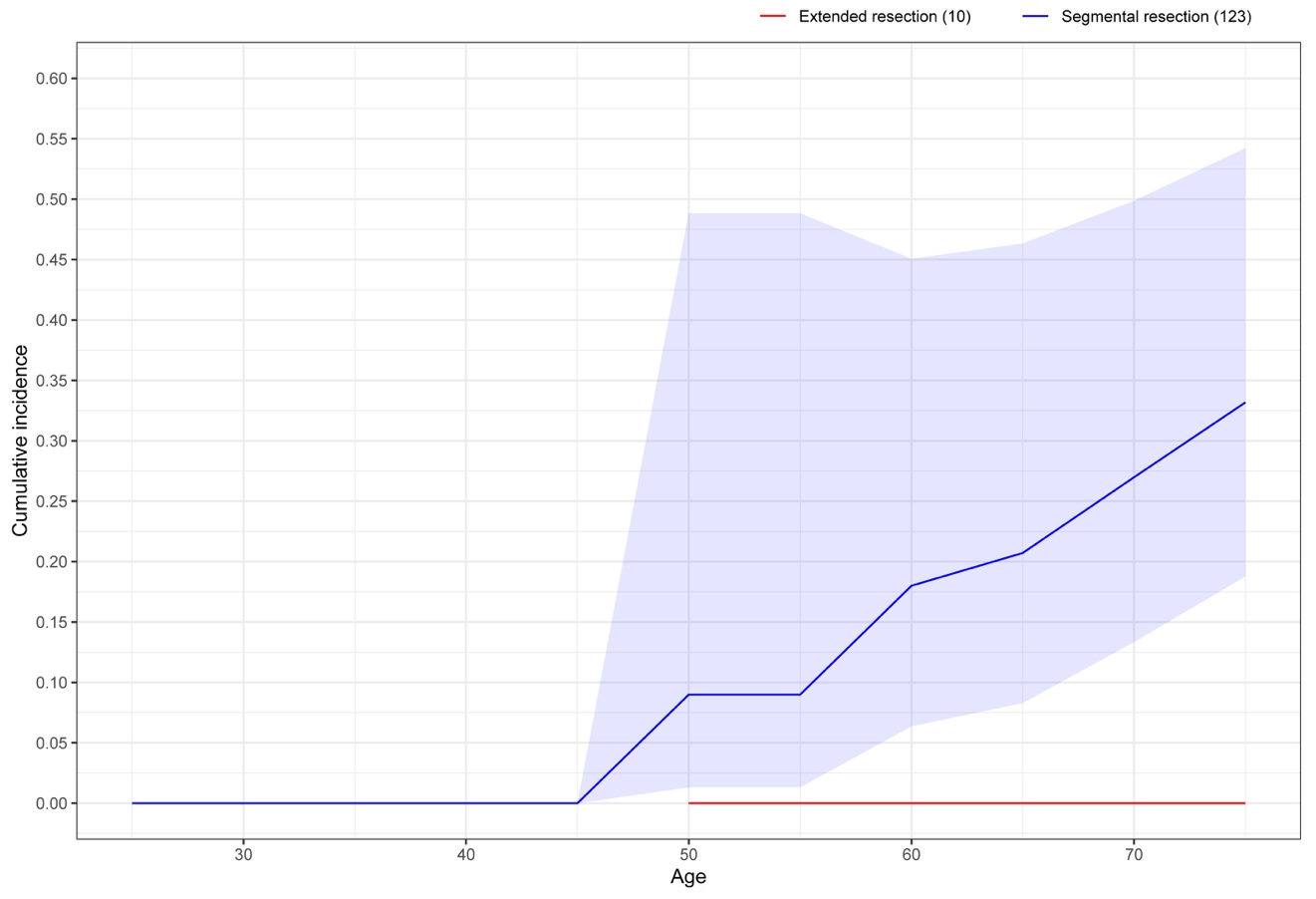


**Figure S2** Cumulative incidence of **metachronous CRC** for *path_MMR* carriers that previously underwent segmental (blue) or extended (red) resection for prior or prevalent CRC by gene and gender (with 95% CIs). **a** all genes male, **b** all genes female, **c** *MLH1* male, **d** *MLH1* female, **e** *MSH2* male, **f** *MSH2* female, **h** *MSH6* male, **i** *MSH6* female.

**a** all genes male **b** all genes female


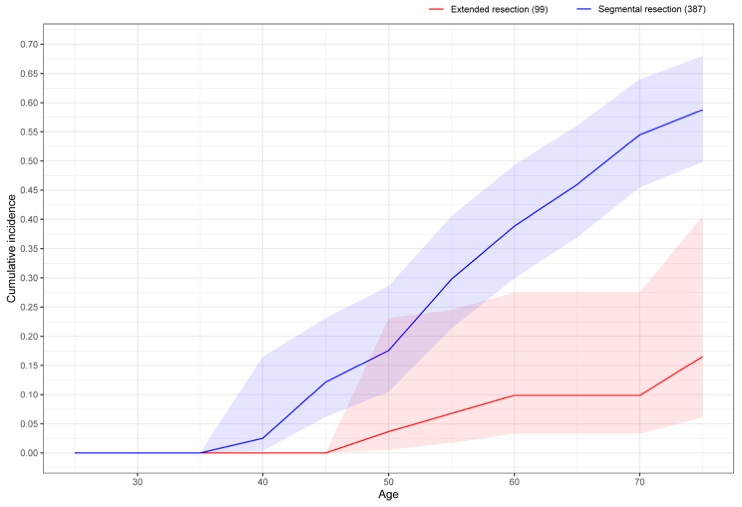

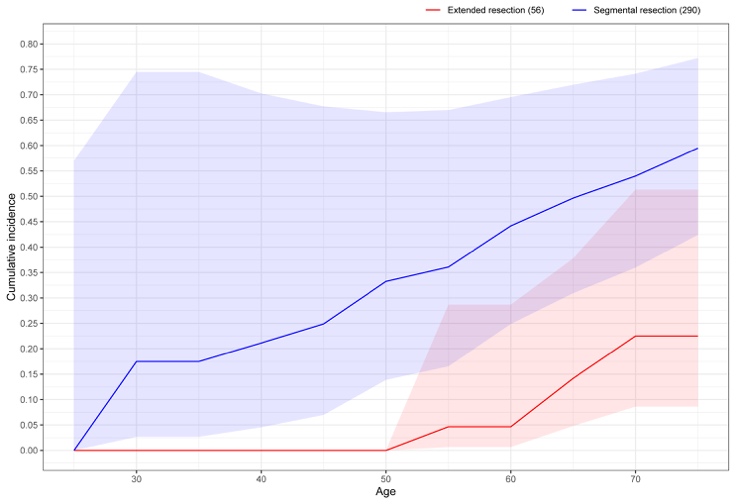


**c** *MLH1* male **d** *MLH1* female


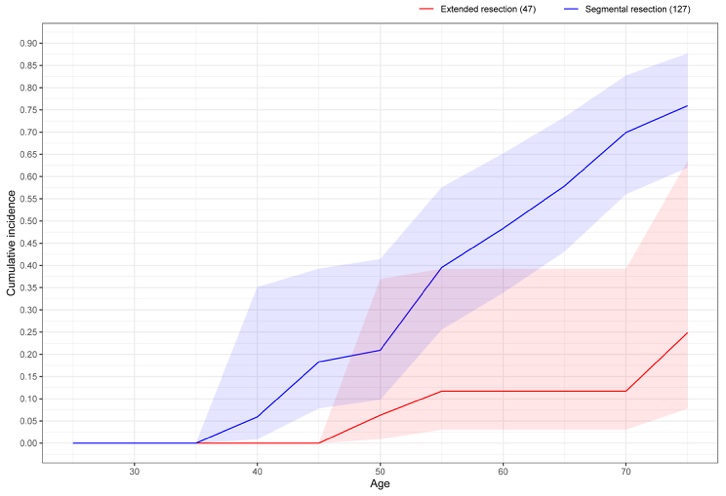

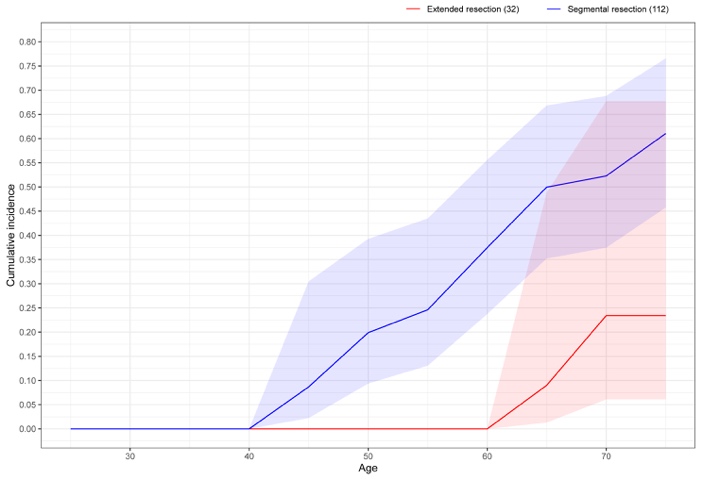


**e** *MSH2* male **f** *MSH2* female


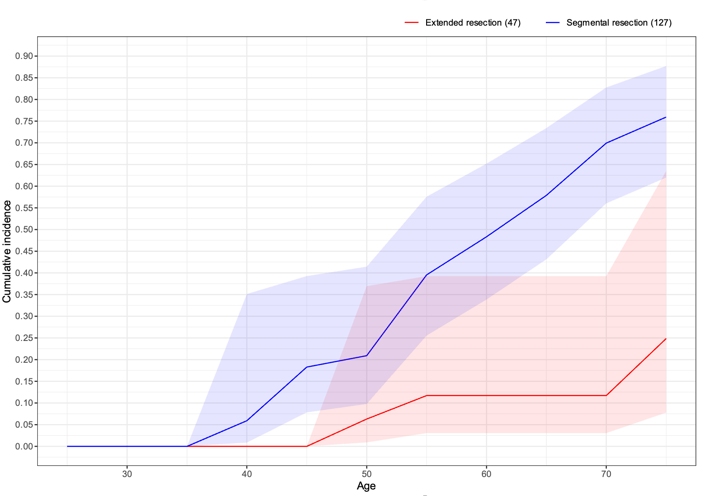

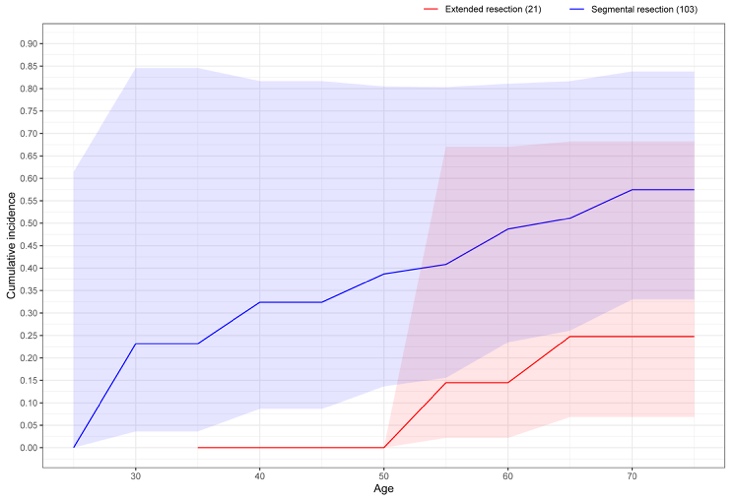


**h** *MSH6* male **i** *MSH6* female


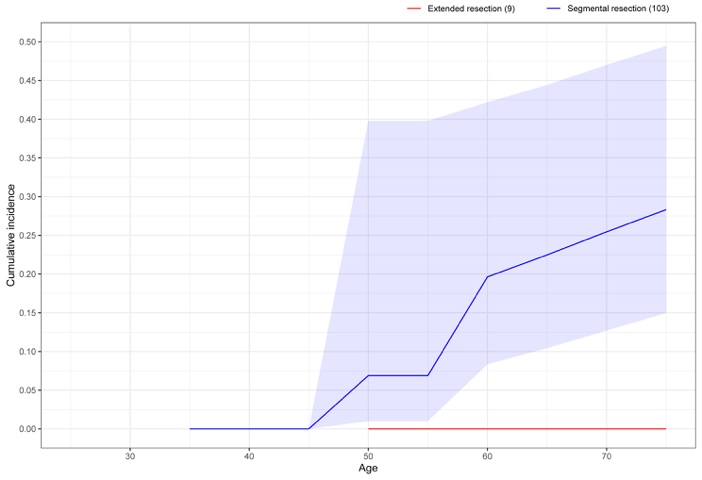

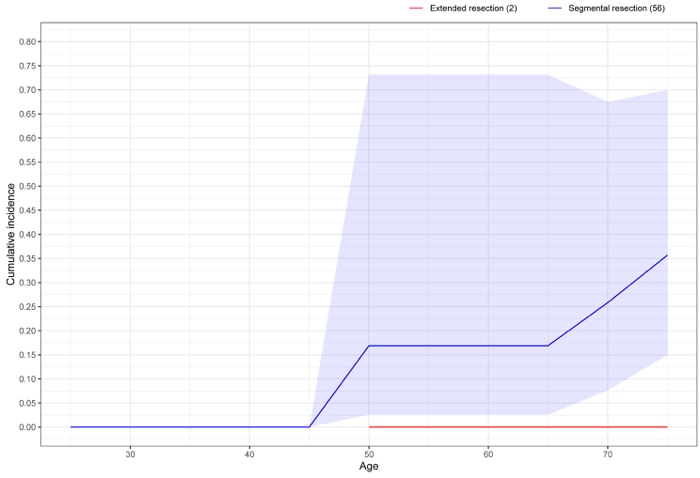


**Figure S3** Cumulative incidence of **metachronous colon cancer** for *path_MMR* carriers that previously underwent segmental (blue) or extended (red) resection for prior or prevalent CRC by gene and gender (with 95% CIs). **a** all genes male, **b** all genes female, **c** *MLH1* male, **d** *MLH1* female, **e** *MSH2* male, **f** *MSH2* female, **h** *MSH6* male, **i** *MSH6* female.

**a** all genes male **b** all genes female


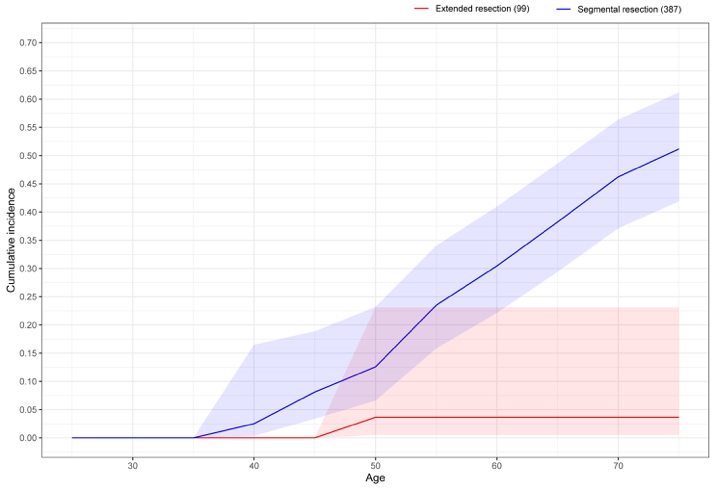

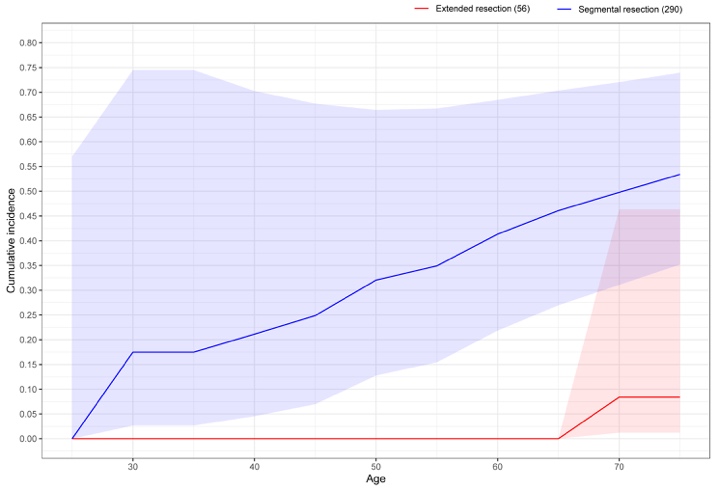


**c** *MLH1* male **d** *MLH1* female


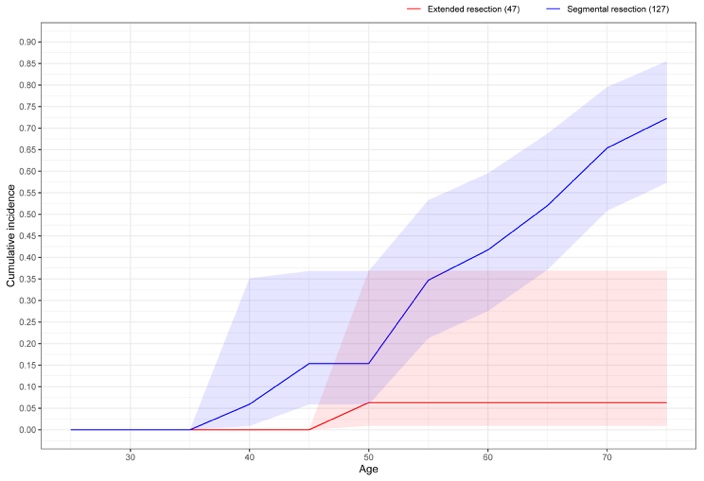

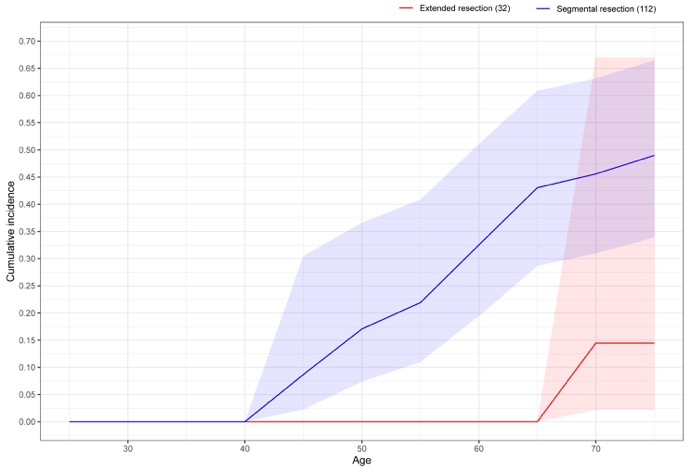


**e** *MSH2* male **f** *MSH2* female


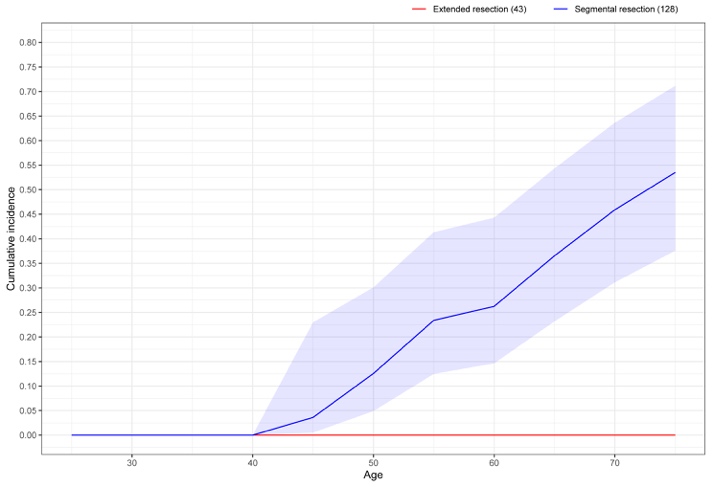

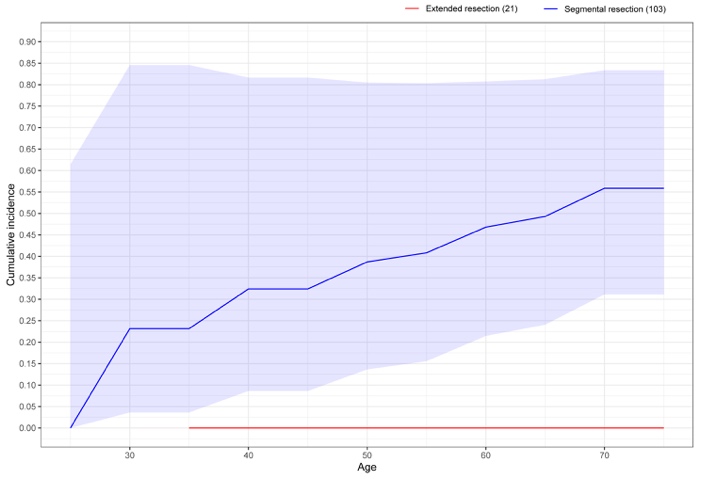


**h** *MSH6* male **i** *MSH6* female


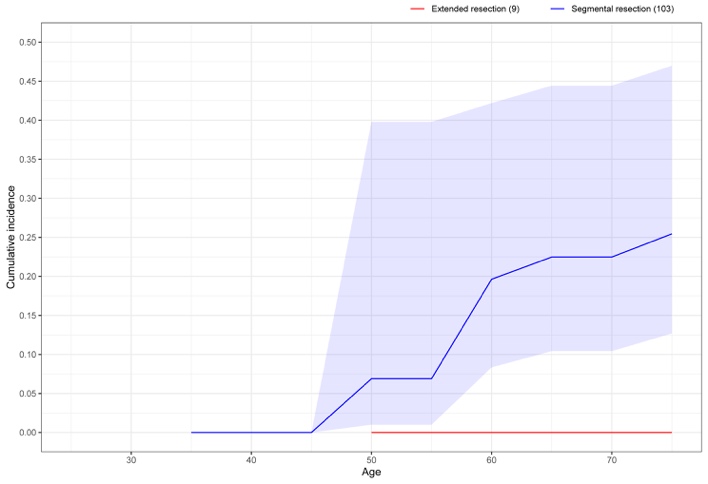

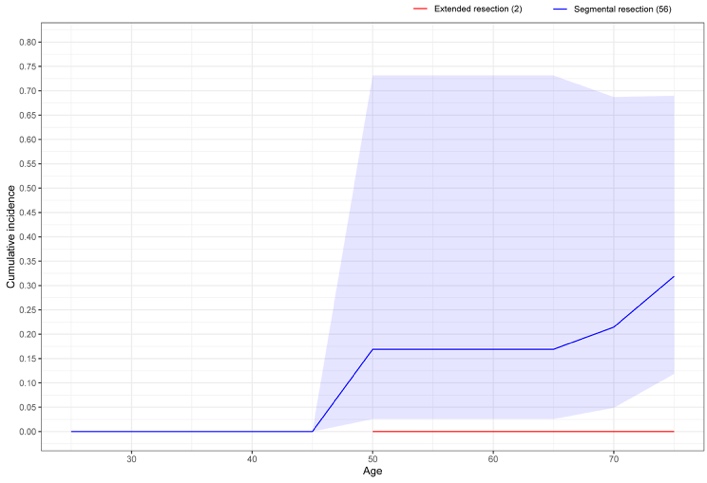


**Figure S4** Cumulative incidence of **metachronous rectal cancer** for *path_MMR* carriers that previously underwent segmental (blue) or extended (red) resection for prior or prevalent CRC by gene and gender (with 95% CIs). **a** all genes male, **b** all genes female, **c** *MLH1* male, **d** *MLH1* female, **e** *MSH2* male, **f** *MSH2* female, **h** *MSH6* male, **i** *MSH6* female.

**a** all genes male **b** all genes female


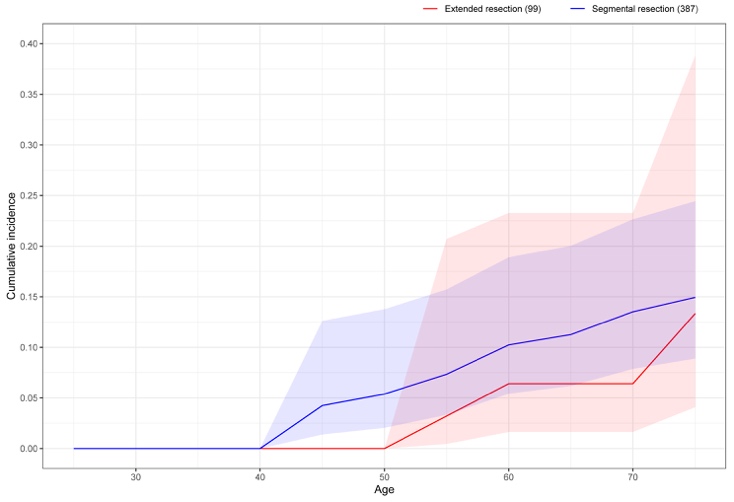

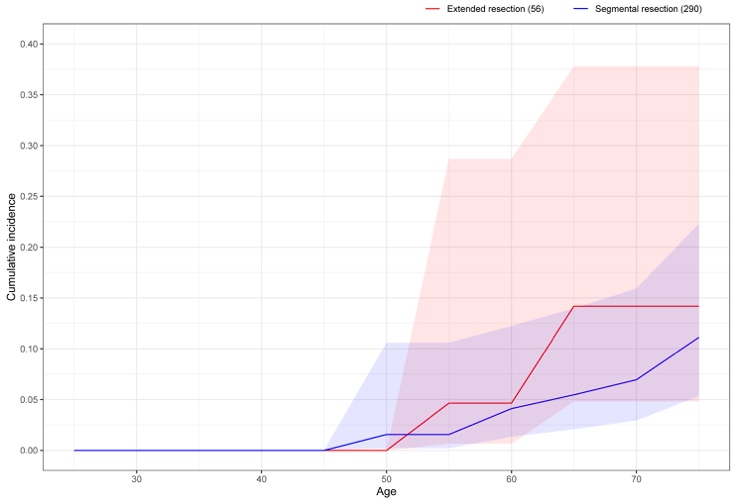


**c** *MLH1* male **d** *MLH1* female


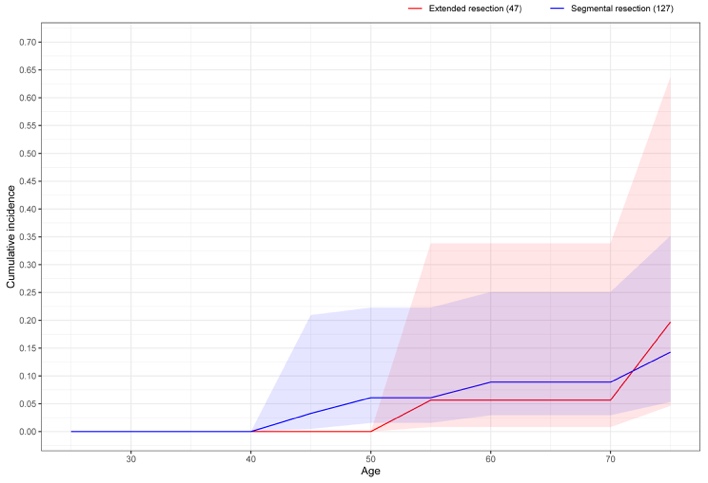

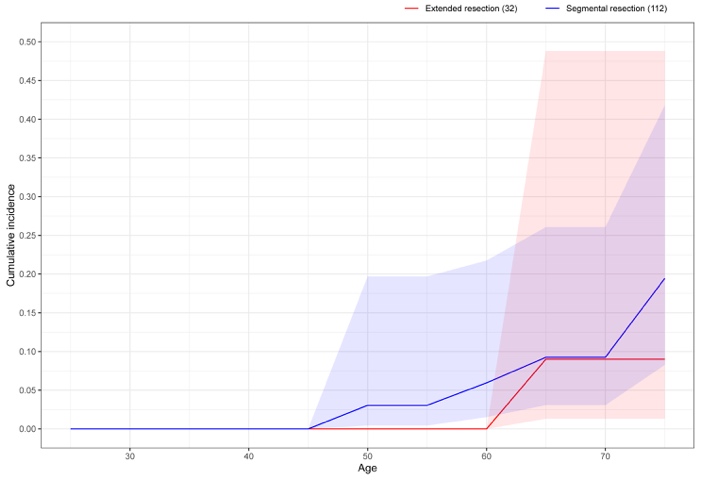


**e** *MSH2* male **f** *MSH2* female


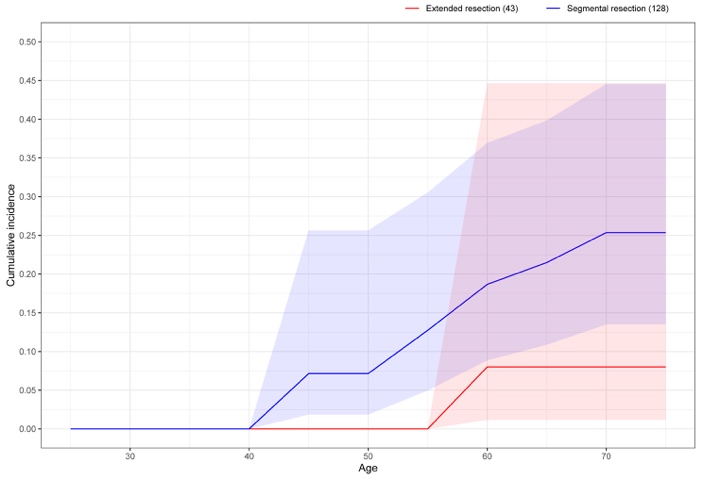

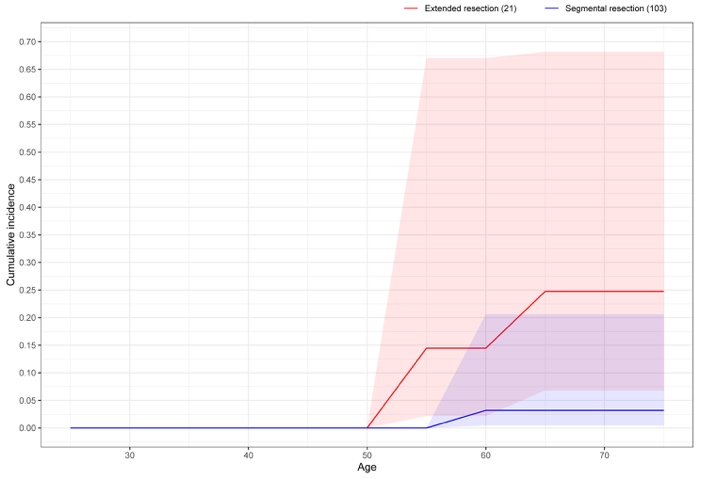


**h** *MSH6* male **i** *MSH6* female


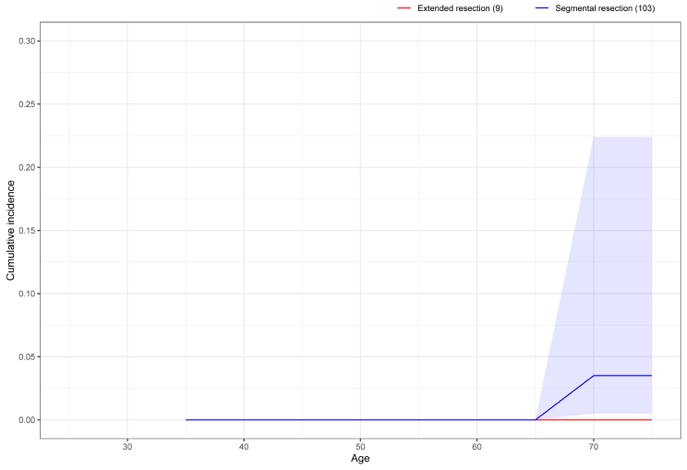

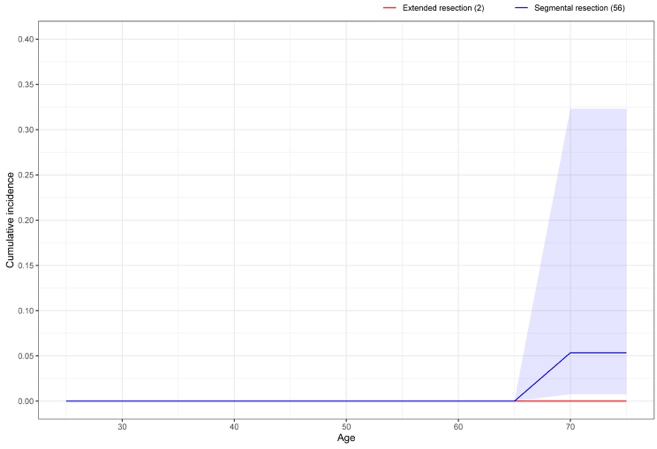


**Figure S5** Cumulative incidence of metachronous CRC for *path_MMR* carriers that underwent right hemicolectomy (red) or left hemicolectomy (blue) for prior or prevalent CRC.

**
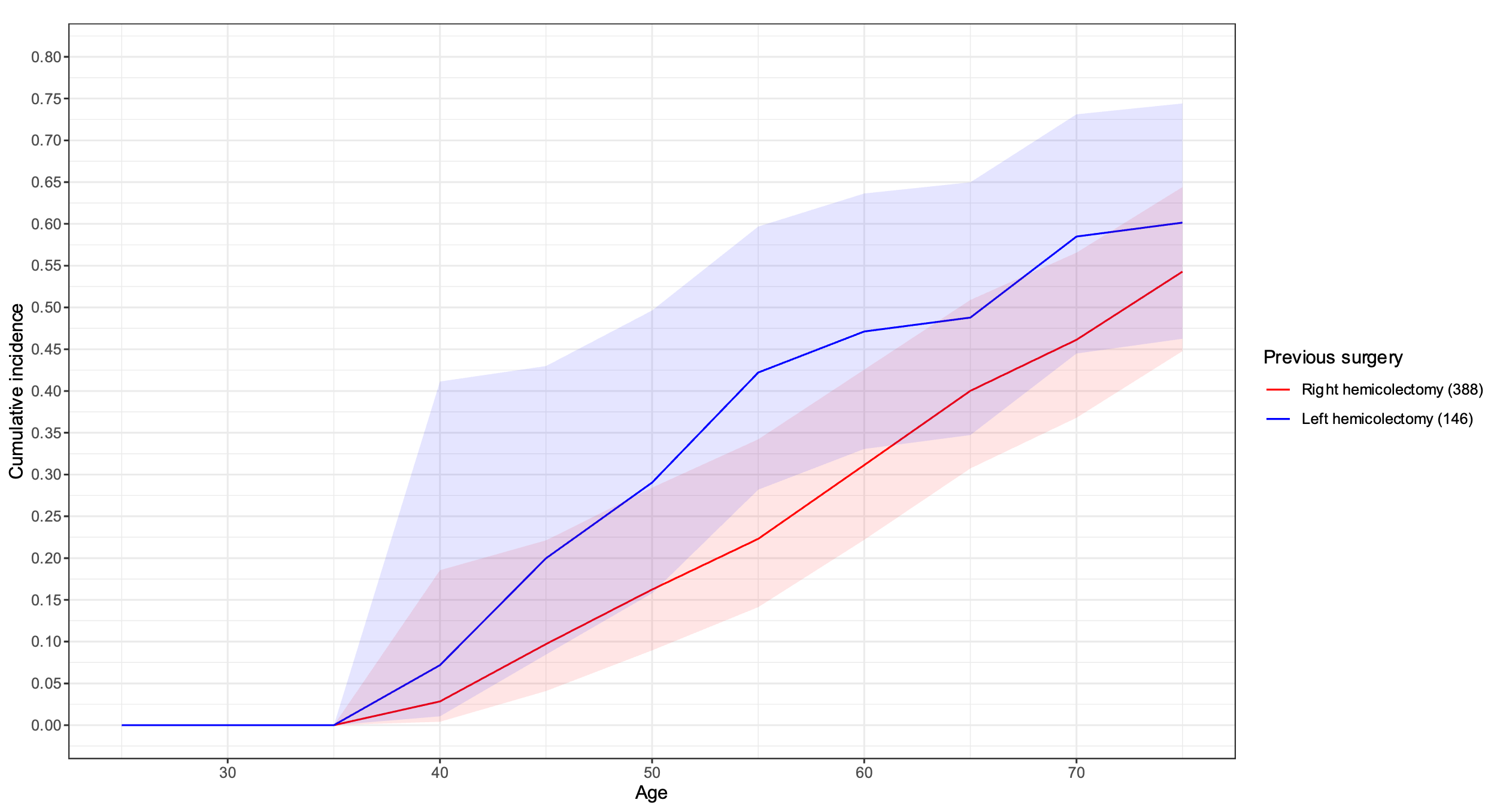
**
